# Methods for implementing a medicine outlet survey: lessons from the anti-malarial market

**DOI:** 10.1186/1475-2875-12-52

**Published:** 2013-02-05

**Authors:** Kathryn A O’Connell, Stephen Poyer, Tsione Solomon, Erik Munroe, Edith Patouillard, Julius Njogu, Illah Evance, Kara Hanson, Tanya Shewchuk, Catherine Goodman

**Affiliations:** 1 Population Services International (PSI), Malaria & Child Survival Department (MCSD), Whitefield Place, Westlands, PO Box, Nairobi, 14355-00800, Kenya; 2 Department of Global Health and Development, London School of Hygiene and Tropical Medicine, 15-17 Tavistock Place, London, WC1H 9SH, UK

**Keywords:** Medicine, Retail outlet survey methods, Price, availability, Market share, Health facility survey, Private sector

## Abstract

**Background:**

In recent years an increasing number of public investments and policy changes have been made to improve the availability, affordability and quality of medicines available to consumers in developing countries, including anti-malarials. It is important to monitor the extent to which these interventions are successful in achieving their aims using quantitative data on the supply side of the market. There are a number of challenges related to studying supply, including outlet sampling, gaining provider cooperation and collecting accurate data on medicines. This paper provides guidance on key steps to address these issues when conducting a medicine outlet survey in a developing country context. While the basic principles of good survey design and implementation are important for all surveys, there are a set of specific issues that should be considered when conducting a medicine outlet survey.

**Methods:**

This paper draws on the authors’ experience of designing and implementing outlet surveys, including the lessons learnt from *ACTwatch* outlet surveys on anti-malarial retail supply, and other key studies in the field. Key lessons and points of debate are distilled around the following areas: selecting a sample of outlets; techniques for collecting and analysing data on medicine availability, price and sales volumes; and methods for ensuring high quality data in general.

**Results and conclusions:**

The authors first consider the inclusion criteria for outlets, contrasting comprehensive *versus* more focused approaches. Methods for developing a reliable sampling frame of outlets are then presented, including use of existing lists, key informants and an outlet census. Specific issues in the collection of data on medicine prices and sales volumes are discussed; and approaches for generating comparable price and sales volume data across products using the adult equivalent treatment dose (AETD) are explored. The paper concludes with advice on practical considerations, including questionnaire design, field worker training, and data collection. Survey materials developed by *ACTwatch* for investigating anti-malarial markets in sub-Saharan Africa and Asia provide a helpful resource for future studies in this area.

## Background

The World Health Organization (WHO) defines its vision for essential medicines as “… that people everywhere have access to the essential medicines they need; that the medicines are safe, effective, and of good quality; and prescribed and used rationally” [1]. In line with this, the Millennium Development Goals acknowledge the need to improve the availability of affordable quality medicines for the world’s poor [2]. Modern medicines account for 20–60% of health spending in developing countries and up to 90% of the population in developing countries purchase medicines through out-of-pocket payments [1]. The WHO Model List of Essential Medicines includes, among other classes of medicines, anti-malarials, anti-bacterial medicines, anti-tuberculosis medicines, anti-retrovirals, oral rehydration salts, vaccines, and contraceptives. Effective prevention and treatment requires that these essential medicines be available at affordable prices at all times [2].

In recent years an increasing number of public investments have been made to improve access to high quality essential medicines by increasing medicine availability and reducing price [3]. Interventions include regulatory measures to ban harmful or expired drugs [4]; policy changes to promote the use of less expensive generics over originator brands; updating or establishing national Essential Medicines Lists to guide public sector procurement and supply; schemes that promote local medicine production; and increased efforts to work with private medicines sellers [5,6]. Given the financial investments and policy maker commitments to increasing access to essential medicines, it is important to monitor the extent to which these interventions achieve their aims using rigorous quantitative data. The impact of these interventions needs to be considered on both the supply of and demand for essential medicines.

It is generally acknowledged that medicine demand has been studied in greater detail than medicine supply. The collection of nationally representative quantitative demand-side data on a broad range of population health topics has been conducted since 1984 through the Demographic and Health Surveys (DHS) and later the Multiple Indicator Cluster Surveys (MICS). This information can be used to answer questions such as where people seek treatment and how quickly, what type of treatment they receive, and how good caregiver knowledge is on causes and symptoms of illness. Many resources are publicly available for such surveys, including sample questionnaires, guidelines for sampling, field worker training materials and data analysis plans [7].

A number of quantitative methods are available to researchers interested in the supply side of the medicine market. The quality of the provider-client interaction can be investigated using mystery shoppers, exit interviews, and observational studies [8]; drug samples can be collected to investigate the prevalence of counterfeit and /or sub-standard medicines in the market [9]; and medicine outlet surveys (sometimes called distribution surveys or retail surveys), can be conducted to collect data on self-reported practices of providers and availability and price of medicines. Medicine outlets are defined as providers that sell/dispense drugs directly to consumers.

However, there are a number of challenges related to studying the supply side of the market, including outlet sampling, gaining provider cooperation and collecting accurate data. In many countries, medicine sellers are not registered and therefore official lists of providers cannot be used as a reliable sampling frame as they may be out of date or exclude important types of medicine sellers such as drug stores, mobile providers or general retailers [10]. Gaining cooperation of providers when information may be legally or commercially sensitive can result in high rates of refusal or under-reporting of products they know to be banned or for which they do not hold a license. Providers may be reluctant to share their records, fearing the information may be disclosed to drug registration bodies or revenue authorities or competitors. Obtaining information related to medicine price, availability and sales volumes is a challenge when multiple brands, formulations and strengths exist. Furthermore, the private commercial sector in many developing countries lacks routine health information system, including routine information on stock. Despite (or more likely, because of) the challenges associated with studying the informal sector, this area has been highlighted as a priority for further methodological development [8].

This paper provides guidance on addressing these challenges when conducting a medicine outlet survey in a developing country context, drawing primarily on previous experience of studying the anti-malarial market. While the basic principles of good survey design and implementation are important for all surveys, the paper highlights a set of specific issues that should be considered when conducting a medicine outlet survey.

## Methods

This paper draws on the authors’ experience of designing and implementing outlet surveys, including the lessons learnt from *ACTwatch* outlet surveys [11] and other key studies in the field. The authors experience was primarily with surveys of the market for malaria-related commodities, but the authors also drew on examples of broader medicine surveys. The *ACTwatch* project investigates markets for anti-malarials and malaria diagnostic testing, and its outlet survey methodology has been implemented more than 30 times since 2008 in over 10 developing countries across Africa and Southeast Asia [11]. Through informal group discussions the authors reflected on their choice of methods, and their experiences in the field, in analysis, and in results dissemination. Key lessons and points of debate are distilled around the following areas: outlet inclusion criteria and appropriate sampling methods; techniques for collecting and analysing data on medicine availability, price and sales volumes; and methods for ensuring high quality data. Where relevant, reference is made to a suite of *ACTwatch* tools that are available online to facilitate further studies in this area.

## Results

### Selecting a sample for a medicine outlet survey

Key study design decisions include selection of outlet types to include, and choice of methods for locating and selecting outlets. The choice of outlet types included in a survey will depend on the nature of the research question, and the study’s aims and objectives. Some studies focus on specific outlet types, such as licensed pharmacies [12], private not for profit clinics [13], or public health facilities [14], while others have a broader remit covering all outlets in the private sector [15,16] or across all sectors where a specific class of medicines may be stocked [17-19]. For example, *ACTwatch* included all outlets with the ‘potential’ to sell anti-malarials, covering public/not for profit and private sectors, and a broad range of outlet types, from public facilities, private hospitals and pharmacies to more informal outlets such as drug stores, kiosks and hawkers [11]. More focused inclusion criteria may lead to a more logistically feasible survey, and provide more detailed data on specific outlet types. However, a comprehensive approach such as that used by *ACTwatch* has the benefit of providing data on the whole market for these medicines, enabling presentation of representative data on all sellers, and calculations of market share, which can be used to monitor the impact of interventions over time. It also allows comparisons between sectors and outlet types. Data on unregistered providers can also be particularly illuminating for policy makers in countries with weak regulatory environments and poor information systems in the private sector.

A variety of methods can be used to develop a sampling frame of outlets. Common approaches include using pre-existing lists, local key informants, and/or a census approach. Depending on the research question one of these methods may be sufficient on its own, however a combination of methods may be required to ensure the most accurate number, and location, of eligible outlets are identified. Official lists provide an easy way of estimating the number of providers operating in a given area by outlet type [12,14]. However, they may be incomplete or outdated (e g, omitting providers who are unregistered or awaiting official registration, or including those whose registration has expired), and generally do not capture provider types operating without authorization, which often make up a large share of medicine sellers [10,15,16]. Privately compiled outlet lists such as those developed during other research studies or by other private institutions (e g, IMS health), may include outlets not captured by official lists [20]. However, such lists may not be available across all settings, and as with official lists, may be outdated. This reflects the high turnover in retail outlets, especially smaller stalls and kiosks. Moreover, given the lack of road maps, or unique identifiers for many outlets, those listed may be difficult to locate. The feasibility of using lists developed by other researchers will also depend on whether consent for sharing these data was obtained at the time of data collection. There may also be a financial charge for such data if they are held by commercial organizations. The use of lists must, therefore, generally be complemented by other outlet identification methods.

Interviews with local key informants (such as people living and working in the study area, community and village leaders and local administrative councils), can be used to update existing lists of medicine sellers, as well as helping to define administrative boundaries and identify the range and location of local outlets selling medicines [12,15,16]. However, such interviews are unlikely to provide a complete and accurate list of all medicines sellers, and therefore also require supplementation.

A census involves a team of data collectors moving systematically through a defined area in order to identify all eligible outlets [10,15,16]. This is likely to be the most comprehensive approach to developing a sampling frame but faces a number of practical implementation challenges. Interviewers must visit all outlets that may meet the study’s inclusion criteria. Where this is based on stocking of certain types of medicines it may be unclear beforehand which types of outlets this will include, necessitating visits to not only clinics, pharmacies and drug shops, but also general stores, market stalls, kiosks etc., which requires significant time and resources. At times, thousands of outlets may be visited but only a small percentage will have medicines in stock. There are also challenges in monitoring the quality of the census to ensure that interviewers approach all relevant outlets. Finally it is only feasible to use the census approach in relatively small geographical areas such as wards or sub-locations.

Once the types of outlet to include in the survey and method for developing the sampling frame has been decided, a number of approaches may be used for sampling outlets. Some studies employ a convenience method, with outlets purposively selected given their proximity to urban or rural centres [18,19]. The most widely used of these approaches is the WHO/HAI methodology, described in Additional file 1. While such an approach can have the benefits of logistical feasibility and low cost, it does not provide a representative sample and may therefore be subject to bias. Where a reliable sampling frame has been obtained, simple random sampling may be used [12], or the sample may be stratified to ensure that sufficient numbers of specific outlet types are captured [14,21]. Alternatively, if a census is conducted to develop the sampling frame, it may be most efficient to include all censual outlets in the sample and complete the full interview at the time of the census [15,16].

In *ACTwatch* the aim was to obtain nationally representative estimates of anti-malarial availability, price and market share. A stratified random sample of local administrative areas (e g, wards, communes, parishes) was selected, with probability proportional to population size (PPS). Stratification was based on characteristics likely to affect key outcomes, for example, urban/rural location, as this was expected to be associated with differences in the reach of supply chains, proximity to markets, and intensity of demand. Sample size was based on estimating the primary indicator (proportion of outlets with any artemisinin-based combination therapy (ACT) in stock among all outlets stocking anti-malarials) with a specified precision [10], with sample size calculations refined in later surveys based on baseline data.

**Figure 1:**
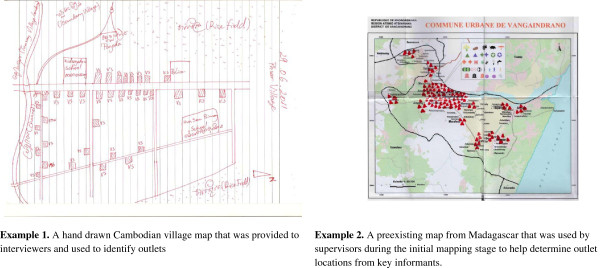
Examples of maps used during field work to identify boundaries and outlets.

Given that many eligible outlets were unregistered, mobile, or recently opened, the census approach was used to develop the sampling frame, supported by the use of key informant interviews with local officials, local maps, and lists of registered outlets where available. Where necessary the data collection team worked with key informants to produce a sketch map to help assign data collectors to specific areas or worked with pre-existing map boundaries to highlight known outlets (see Figure 1 for an example). In addition, the snowball technique was used, asking providers interviewed if they could identify other outlets like their own in the area where they operated. All outlets visited were screened to assess eligibility and only those with anti-malarials in stock or who had stocked them in the previous three months were invited to participate in the survey.

The *ACTwatch* experience highlighted two outlet types that were particularly challenging to identify: itinerant vendors and community health workers (CHWs). In keeping with inclusion criteria for other outlet types, itinerant vendors (also known as hawkers) were only considered as having the potential to sell anti-malarials if they were hawking general household goods, medicines and/or soap. Hawker congregation points were identified using key informant interviews and hawkers were approached by interviewers and asked if they had already participated in the survey to avoid duplication. For interviewer safety, hawkers were only approached during daylight hours, given these vendors work late into the night and in potentially unsafe areas.

Across countries, there is considerable variation in the names used for CHWs, their roles, funding source, and registration status. While some CHWs administer anti-malarials, others work for health interventions unrelated to malaria; therefore decisions on the possible inclusion of CHWs in the census must be country specific. When available, registered listings may be used to locate CHWs in the field. For example, in some countries there are links between health posts and CHWs, and updated lists of CHWs can be provided through the health post providers. Alternatively there may be an elected CHW representative who can be contacted for updated lists. CHWs can sometimes be identified within a community due to sign-posts outside their homes.

There is also an important temporal element to sampling, as key outlet characteristics can be expected to change seasonally in response to fluctuations in disease burden and accessibility.

Wherever possible ACTwatch surveys were conducted during the months of peak malaria transmission in each country, and follow-up surveys repeated during the same months in subsequent years. Where this was not possible, surveys were conducted shortly after the peak transmission season. For example, in Madagascar, seasonal peaks in malaria transmission coincide with the months when parts of the country are inaccessible due to heavy flooding and the presence of cyclones, making it difficult to undertake fieldwork which was, therefore, scheduled just after the months of peak transmission.

### Collecting data on medicines

#### Collecting availability, price and sales volume data

Most medicine surveys include a set of questions on the characteristics of the outlet and the respondent such as number of staff, qualifications and provider knowledge. These questions can be considered relatively straightforward in comparison with collecting data on medicines. Collecting survey data on the latter is highly complex due to the wide variation in products by generic type, formulation, strength, and pack sizes, and differences in outlet practices. The first issue to consider is whether data collected will be restricted to a predetermined checklist of medicine brands, or more open ended allowing collection of data on a wider range of medicines. Using a standard predetermined list can facilitate comparison across countries and also simplify data collection as field workers are not required to categorize medicines during the interview (see for example the WHO/HAI method described in Additional file 1). The WHO reference list of essential medicines, for example, can be used as pre-determined list. Medicines can be pre-coded in advance if using PDAs or other electronic capture systems. However, using a more open-ended approach means data can be collected on all products within a therapeutic class, thus allowing the calculation of market share, and highlighting availability of non-recommended or banned products, whose presence might not have been expected. An open-ended approach may also be more flexible where there is a very wide variety of potential brands and pack sizes.

In collecting price data, clarity is required on whether data should be collected per pack/tin or per unit (e.g., tablet, ampoule). This is important to consider given that, for example, tablet medicines may be stored in tins of 100 or 1000 tablets or as pre-packaged therapies of 24 tablets for an adult course. In addition, clear guidance should be given on the handling of any non-drug costs that are incurred, for example for registration, consultation, syringes or diagnostic testing, which may only be required at some outlet types (eg, health facilities but not drug shops). In including these costs one could be accused of not comparing like with like, but in excluding them one may misrepresent the true costs to patients of visiting alternative outlet types. Ideally they should, therefore, be collected so that the analysis can be done with and without such associated costs. Care should also be taken to collect the retail rather than wholesale sale price of medicines in outlets that function in both capacities, which may be common for larger pharmacies that wholesale medicines to smaller pharmacies or drugs stores. Finally, where exemption mechanisms exist for certain age groups in, for example public health facilities, such exemptions can be noted in the comment fields of the questionnaire. Decisions on how to treat this can then be dealt with at the analysis stage.

As with price data, clear guidance must be given on the units for collection of sales volumes (e g, tablets *versus* packs *versus* boxes). The collection of sales volume data is further complicated by the need to record sales over a previous time period. One approach is to collect information from sales records [22], but in practice these are frequently inaccurate, or in the private sector, not kept at all, or viewed by sellers as confidential. Alternatively, outlet staff can be asked to recall their sales volumes during the week or two weeks preceding the interview [10]. As with all survey recall questions, recall bias may be a problem if recall is worse of sales conducted further into the past. However, limiting the recall period to one or two weeks does not capture seasonal variation of medicine sales (for example, fluctuations caused by higher or lower malaria transmission seasons) and longer-term stock-outs. A third approach is to undertake a retail audit where information is collected by field teams visiting a panel of outlets at regular intervals [15,20,23,24]. At each visit and in each outlet field workers measure the stocks of each product and ask about any volumes added and/or disposed of during the visit interval, in order to estimate sales. This method avoids the need to use outlet records or rely on recall of sales volumes, but still requires recall of wholesale purchases and stock disposed of, and can be challenging where sellers resist having their stock counted. This method also imposes considerable time and cost burdens on the data collection team, and may lead to respondent fatigue [23].

### Generating comparable price and sales volume data: the adult equivalent treatment dose

A challenge in conducting medicine outlet surveys is the measurement of price and sales volume in a standardized way across drug types because of the considerable variation in strength, pack size, formulation and dose length across products, even within a given therapeutic class. Moreover, while some drugs are pre-packed for specific age groups, others are sold loose, eg, from pots of 500 or 1,000 tablets. For chronic conditions, measures such as the “defined daily dose” have been used as the unit of comparison [25], but this is inappropriate for short-term treatments where treatment duration varies [26]. For example, it would not be appropriate to compare data for a daily dose of the anti-malarial sulphadoxine-pyrimethamine with quinine, given that the former is a one-day treatment regimen and the latter is seven days. An alternative used for anti-malarials is the adult equivalent treatment dose (AETD) [5,10,24,26,27]. One AETD is defined as the number of milligrams (mg) of an anti-malarial ingredient needed to treat a 60 kg adult. Under *ACTwatch*, for each anti-malarial category, the number of mg in one AETD is defined as that recommended in the treatment guidelines for uncomplicated malaria in areas of low drug resistance issued by the WHO [10]). Where WHO treatment guidelines do not exist, an AETD is based on peer reviewed research, or where this is not available, on the product manufacturer’s recommended treatment course. In the case of ACT, with two or more active anti-malarial ingredients packaged together, the strength of the artemisinin-based component is used as the basis for the AETD calculations.

There are some cases when one AETD equates precisely to a ‘real’ product. One example is the Coartem® 24 tablet package. In total the 24 tablets contain 480 mg artemether, which is the amount of artemether that defines one AETD (Table 1). By extension, a 12-tablet pack equals 0.5 AETD, and a 6-tablet pack equals 0.25 AETD.

**Table 1:** Common adult equivalent treatment dose (AETD) reference values for anti-malarial active ingredients

**Generic name**	**Ingredient used for AETD calculation**	**mg dose required for 1 AETD**	**Source**
Quinine	Quinine	10408 (as base^1^)	WHO Model Formulary, 2008
Sulphadoxine-pyrimethamine	Sulphadoxine	1500	WHO Model Formulary, 2008
Artemether-lumefantrine	Artemether	480	WHO Model Formulary, 2008
Artesunate-Amodiaquine	Artesunate	600	Manufacturer recommendations (also quoted in the WHO Guidelines for the treatment of malaria, 2^nd^ edition, 2010)

^1^ Due to the pharmacodynamic properties of active ingredients, some medicines are manufactured as salts in order to improve the taste or slow down the absorption of the active ingredient. Care needs to be taken with such products as the strength in salt form will be greater than the base strength of the active ingredient. For example, 10,408 mg of quinine base corresponds to 12,600 mg of quinine sulphate, a common salt used in the presentation of quinine tablets.

Source: *ACTwatch.*

The minimum attributes on each product needed to conduct AETD calculations are show in Table 2. It may also be useful to collect additional attributes such as brand name, manufacturer and country of manufacture to assist with data cleaning, assess market share by manufacturer and country of origin, and allow drugs to be classified into categories such as WHO-approved or nationally registered.

**Table 2:** Medicine attributes essential for the correct calculations of adult equivalent treatment doses (AETDs)

**Attribute**	**Role in AETD calculation**	**Notes**
Generic name	Used to define the number of milligrams of active ingredient required for 1 AETD.	Also enables classification of medicines by different classes for analysis (e g, monotherapies *vs* combination therapies)
Strength of active ingrediets	Taken together, strength and pack size are used to calculate how many milligrams of active ingredient are present in the medicine package.	Also enables classification of medicines, most notably used to flag first-line treatments, which are defined in terms of generic name and strength.
Pack size		Requires different definitions for tablet and non-tablet medicines.
Is product a fixed dose combination?	One of these two attributes is required in order to establish the ratio of tablets in co-blistered medicines. This information is then used to modify the pack size value in the AETD calculation, if necessary.	
Brand name		

Source: *ACTwatch.*

It should be noted that where many customers are children and where purchase of under-doses is common, the estimated number of AETDs sold will be lower than the actual number of sales made. Similarly, the average price per AETD will be higher than the average price paid per customer. Furthermore the AETD approach can mask differences in costs between adult and paediatric packs as all products within a generic category are generally combined to estimate price per AETD. While an advantage of the AETD approach is that it allows comparison of prices across formulations (tablets *versus* syrups *versus* injectables), care should be taken in presenting average price data for several formulations combined as the price distributions for non-tablet formulations tend to be different from those for tablets, with much higher medians, implying that it could be misleading to use one measure of central tendency across different formulations [10]. The AETD approach could be extended to calculate price and sales volumes for other medicines, though it is less appropriate for drugs that are not taken in a course of a specific length, e g, paracetamol.

### Ensuring high quality data collection in a medicine outlet survey

Key quality assurance steps common to all research studies include questionnaire design and field worker training. An additional area that may require particular attention for medicine outlet surveys is identifying all relevant medicines in an outlet.

### Questionnaire design

**Figure 2:**
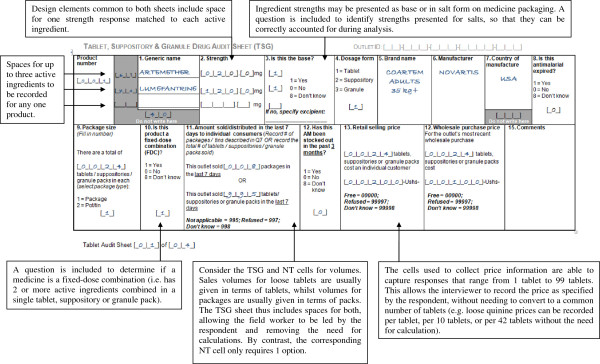
**Examples of the tablet, suppository and granule (TSG) audit sheets from the *ACTwatch *questionnaire.**

**Figure 3:**
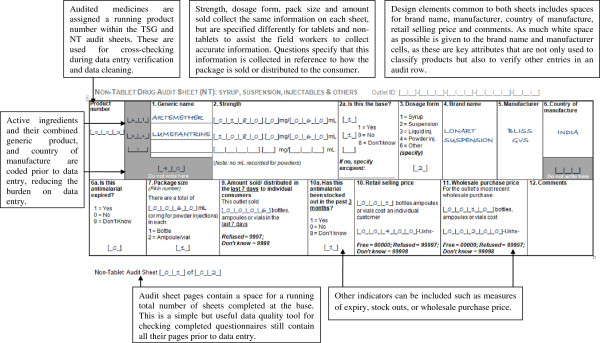
**Examples of the non-tablet (NT) audit sheets from the *ACTwatch *questionnaire.**

Good questionnaire design can help greatly to reduce errors during fieldwork, particularly in the design of “audit sheets” for collection of data on products stocked. *ACTwatch* audit sheets represent a useful resource for future surveys given that they have been refined based on the experience of numerous survey rounds [28]. They have the following key features:

Given the different data required, separate audit sheets are use for “tablet, suppository and granule products”, and syrup and injectable or “non-tablet” products (see examples in Figures 2 and 3);

Audit sheets are designed to allow entry of combination medicines with up to three active ingredients;

A comments box is provided to allow data collectors to note anything unusual about the product which may affect the calculation of AETDs (e g, if an anti-malarial is packaged with non-anti-malarial and a correction to the recorded pack size required during analysis. In some instances Fansidar (SP) is packaged with paracetamol and interviewers should record the number of tablets of each product in the comments;

The sheets allow flexibility on the units for which prices and volumes are recorded, avoiding the need for data collectors to perform calculations themselves;

Additional audit sheets can be added if required in well-stocked outlets;

Each product and page has a serial number and the number of audit sheets completed/medicines are recorded on the main body of the questionnaire to ensure that all are present during data entry.

### Field worker training

For a medicine outlet survey, ensuring quality of training and thorough comprehension by field workers can be more challenging than for other surveys because of the detailed medicine-related data collected. However, in common with all surveys, good training is the key to success. *ACTwatch* have developed a seven-day outlet survey training curriculum that details how to complete each section of the questionnaire, as well as covering conducting a census and good interviewing practice, and including practice in the field (Additional file 2). A full facilitator curriculum is provided, including presentation slides for each topic and advice on points to be made with each slide. The sessions are highly interactive, involving numerous practical exercises and games to emphasize key concepts, and a series of interviewer tests to assess knowledge throughout the training period. Repetition is built into the schedule, with earlier topics re-examined and tested as new ones are introduced. These training materials, as well as standard operating procedures to help prepare for the training and fieldwork, are available on the web [28]. Training materials can be used with only minor country specific adaptations, for example, to include locally common brands and outlet types, and have been adapted for other anti-malarial surveys.

Lessons learned from successive rounds of *ACTwatch* surveys have fed into the training materials and in particular the approach taken to introduce certain topics. For example, the authors have found it useful to introduce the topics of generic names and brand names with reference to everyday items such as milk or bread; and when discussing the strength of liquid formulations it has been helpful to have a practical demonstration of dissolution in class. Prior to training, a large variety of medicines available in the country are purchased for presentation during training sessions and use in exercises and tests, representing the broadest possible range of dosage forms, brands, generics and pack sizes. Having access to a wide range of examples not only allows the trainers to present many different scenarios during the training sessions, but rotating medicines between field workers during tests helps to mitigate against people ‘remembering’ the correct answers for a given product.

Given the complexity of the training programme, experience shows that despite intensive training not all field workers are able to grasp these issues. It is therefore advisable to over-recruit trainees, to ensure sufficient numbers are eligible for the fieldwork.

*ACTwatch* has also developed additional training materials for team supervisors and quality controllers who conduct back-checks to outlets already visited by data collectors to check on performance.

### Identifying all relevant drugs in an outlet

For a study employing a ‘full audit’ approach for a given medicine or disease class, a major challenge is ensuring that all relevant products are audited. Without due care it is common to find that products are missed because they are forgotten, sellers do not realize that they are relevant, sellers wish to conceal them as they believe they are not permitted to stock them, or interviewers ignore them to reduce the data collection workload. Several strategies can be employed to minimize this.

Firstly, the information provided during consent procedures should make it explicitly clear that the interview is not an inspection and that the data gathered will be confidential in order to encourage full disclosure. Secondly, it is helpful to include a set of prompts in the questionnaire around specific generic types and formulations to reinforce the need to audit *all* medicines, and not just the most common ones. This can be complemented by a photo book of different types of medicines, to remind the seller of any other drugs that might be in stock. Data collectors should also be trained to recognize relevant medicines so they can (subtly) scan the products on display in outlets.

### Quality control checks

Field supervisors should review all completed questionnaires for completeness. Supervisors should also note challenges with specific questions, which interviews may commonly skip or mark incorrectly. In addition, back-check visits to outlets should be conducted by separate quality control staff to check that outlets were indeed visited and to counter any incentives for data collectors to try to reduce data collection time by for example skipping certain products in the audit. With a well-designed back check questionnaire it is not necessary for quality control staff to repeat the whole questionnaire, but rather to collect data on specific key variables. This can also provide an opportunity to ask questions about the interviewer’s conduct and politeness. General observation by supervisors is also key to monitor fieldworkers adherence to fieldwork practices including consent procedures, conducting the census, and use of data collection materials.

## Conclusion

This paper has provided guidance on key steps in conducting a medicine outlet survey in a developing country context, drawing on the *ACTwatch* project and other studies. While the basic principles of good survey design and implementation are highly relevant, there are also a set of specific issues that should be considered when conducting an outlet survey. These include careful consideration of: inclusion criteria by outlet type; methods for developing a reliable sampling frame and for selecting outlets; specific issues in the collection of data on medicines; and approaches for generating comparable price and sales volume data across products. Given the resulting complexity of survey methods it is essential that this is complemented with a well-designed questionnaire, rigorous field worker training and strong quality control procedures. The survey materials developed by *ACTwatch* provide a helpful resource for future studies on anti-malarials and markets for medicines in general.

## Competing interests

The authors declare that they have no competing interests.

## Authors’ contributions

KOC designed the paper and *ACTwatch* study design. KOC, TS, SP, and EP wrote the manuscript. IE, JN, and EM contributed to the *ACTwatch* approaches and examples presented in the manuscript. TS, CG, and KH contributed to the structure and subsequent drafts of the manuscript. CG provided guidance on the manuscript structure. All authors read and approved the final manuscript.

## Supplementary Material

Additional file 1 **Summary of the WHO/HAI approach to outlet sampling.** The World Health Organization (WHO)/Health Action International (HAI) methodology for sampling medicine outlets has been standardized and applied across many countries [29,30]. The surveys aim to monitor to the price, availability and affordability (price) of medicines. The guidelines recommend selecting outlets in six geographic areas across four sectors, namely: public sector hospitals and clinics, the formal commercial sector, unlicensed or informal commercial sector, and not-for-profit organizations (where present). Survey areas include the main urban centre plus three areas randomly selected from those that can be reached within a day’s travel from the main urban centre. In each survey area, the main public hospital, plus four randomly selected public medicine outlets reachable within 4 hours’ travel from the main hospital define the public sector facility sample. The private sector and other sector samples are identified by selecting one medicine outlet in each sector that is geographically closest to each public outlet. This results in a basic sample size of up to 120 outlets [29]. This sampling strategy has been used to provide routine monitoring data for essential medicines across a number of countries [19,31-33]. The WHO/HAI method restricts inclusion to a maximum of 50 medicines. These include 14 global and 16 regional essential medicines, pre-determined by WHO/HAI to enable cross-country comparisons, and 20 supplementary essential medicines identified at the country level. For each of the 50 generic medicines selected, two products are then surveyed: i) the originator brand (using the same pack size and strength in each outlet) and ii) the lowest-priced generic product that contains the same active ingredient as the originator brand. The HAI/WHO method is a highly feasible approach to implement and can be notably less expensive than surveys that implement a census approach. The purposeful selection of sampling areas and outlets however limits the ability to extrapolate findings to the national level, and the relatively small sample size means the method does not have the power to detect small but potentially important differences in indicators. The HAI method has been adapted to overcome this challenge by identifying all private registered and unregistered sources of anti-malarial drugs within three hours’ drive from each public health facility using lists of outlets and key informants, and randomly sampling five outlets of each type [17]. However, the restriction to five outlets per area from each sector risks missing differences between different types of informal commercial outlets in countries that support a diverse informal medicine market. Additional resources, including study design templates and analysis folder in Excel can be found on the HAI website. Click here for file

Additional file 2 **Training topics for *ACTwatch *outlet surveys.** Click here for file
