# Supplementary material for: Methods for implementing a medicine outlet survey: lessons from the anti-malarial market
Source: Malar J. 2013 Feb 5;12:52. doi: 10.1186/1475-2875-12-52 (PMC3599752; doi:10.1186/1475-2875-12-52)
Supplement: Additional file 1 — Summary of the WHO/HAI approach to outlet sampling. The World Health Organization (WHO)/Health Action International (HAI) methodology for sampling medicine outlets has been standardized and applied across many countries [29,30]. The surveys aim to monitor to the price, availability and affordability (price) of medicines. The guidelines recommend selecting outlets in six geographic areas across four sectors, namely: public sector hospitals and clinics, the formal commercial sector, unlicensed or informal commercial sector, and not-for-profit organizations (where present). Survey areas include the main urban centre plus three areas randomly selected from those that can be reached within a day’s travel from the main urban centre. In each survey area, the main public hospital, plus four randomly selected public medicine outlets reachable within 4 hours’ travel from the main hospital define the public sector facility sample. The private sector and other sector samples are identified by selecting one medicine outlet in each sector that is geographically closest to each public outlet. This results in a basic sample size of up to 120 outlets [29]. This sampling strategy has been used to provide routine monitoring data for essential medicines across a number of countries [19,31-33]. The WHO/HAI method restricts inclusion to a maximum of 50 medicines. These include 14 global and 16 regional essential medicines, pre-determined by WHO/HAI to enable cross-country comparisons, and 20 supplementary essential medicines identified at the country level. For each of the 50 generic medicines selected, two products are then surveyed: i) the originator brand (using the same pack size and strength in each outlet) and ii) the lowest-priced generic product that contains the same active ingredient as the originator brand. The HAI/WHO method is a highly feasible approach to implement and can be notably less expensive than surveys that implement a census approach. The purposeful selection of s [file 1475-2875-12-52-S1.doc]

**Figure** 1: Summary of the WHO/HAI approach to outlet sampling

| The World Health Organization (WHO)/Health Action International (HAI) methodology for sampling medicine outlets has been standardized and applied across many countries [22, 23]. The surveys aim to monitor to the price, availability and affordability (price) of medicines. The guidelines recommend selecting outlets in six geographic areas across four sectors, namely: public sector hospitals and clinics, the formal commercial sector, unlicensed or informal commercial sector, and not-for-profit organizations (where present). Survey areas include the main urban centre plus three areas randomly selected from those that can be reached within a day’s travel from the main urban centre. In each survey area, the main public hospital, plus four randomly selected public medicine outlets reachable within 4 hours’ travel from the main hospital define the public sector facility sample. The private sector and other sector samples are identified by selecting one medicine outlet in each sector that is geographically closest to each public outlet. This results in a basic sample size of up to 120 outlets [22]. This sampling strategy has been used to provide routine monitoring data for essential medicines across a number of countries [19, 24-26].  The WHO/HAI method restricts inclusion to a maximum of 50 medicines. These include 14 global and 16 regional essential medicines, pre-determined by WHO/HAI to enable cross-country comparisons, and 20 supplementary essential medicines identified at the country level. For each of the 50 generic medicines selected, two products are then surveyed: i) the originator brand (using the same pack size and strength in each outlet) and ii) the lowest-priced generic product that contains the same active ingredient as the originator brand.  The HAI/WHO method is a highly feasible approach to implement and can be notably less expensive than surveys that implement a census approach. The purposeful selection of sampling areas and outlets however limits the ability to extrapolate findings to the national level, and the relatively small sample size means the method does not have the power to detect small but potentially important differences in indicators. The HAI method has been adapted to overcome this challenge by identifying all private registered and unregistered sources of anti-malarial drugs within three hours’ drive from each public health facility using lists of outlets and key informants, and randomly sampling five outlets of each type [17]. However, the restriction to five outlets per area from each sector risks missing differences between different types of informal commercial outlets in countries that support a diverse informal medicine market. Additional resources, including study design templates and analysis folder in Excel can be found on the HAI website. |
| --- |
