# Supplementary material for: Methods for implementing a medicine outlet survey: lessons from the anti-malarial market
Source: Malar J. 2013 Feb 5;12:52. doi: 10.1186/1475-2875-12-52 (PMC3599752; doi:10.1186/1475-2875-12-52)
Supplement: Additional file 2 — Training topics for ACTwatch outlet surveys. [file 1475-2875-12-52-S2.docx]

**Additional File 2: Training topics for *ACTwatch* outlet surveys**

An indication of the areas covered by the *ACTwatch* training sessions is given below. For almost all topics, hands-on exercises with medicine packages and other materials are used to reinforce learning. Technical training on the audit sheet is broken up by other topics, to provide variety during the sessions.

**Interviewer training**

Overview of health and malaria situation in the country

Overview of *ACTwatch*

Audit sheet: Generic names and brand names

Audit sheet: Medicine formulations

Audit sheet: Pack sizes

Audit sheet: Rapid diagnostic tests for malaria

Outlet types and inclusion criteria

Outlet selection, ethics and consent procedure

Conducting the census

Audit sheet: sales volumes

Audit sheet: retail price

Provider questionnaire review

Roles and responsibilities in the field

Overcoming obstacles in the field

Data collection monitoring sheets

**Supervisor / Quality controller training**

Recap of team structures, roles and responsibilities

Advocacy in the field

Overcoming obstacles

Data integrity: Questionnaire management

Data integrity: Questionnaire review (includes full review of questionnaires from field practice)

Data integrity: Quality control procedures (e g, back-checks)
